# Supplementary material for: Seroprevalence of SARS-CoV-2 antibodies and retrospective mortality in two African settings: Lubumbashi, Democratic Republic of the Congo and Abidjan, Côte d’Ivoire
Source: PLOS Glob Public Health. 2023 Jun 8;3(6):e0001457. doi: 10.1371/journal.pgph.0001457 (PMC10249894; doi:10.1371/journal.pgph.0001457)
Supplement: S1 Text — (DOCX) [file pgph.0001457.s001.docx]

**S1 Text**

**Supplementary information and supplementary analyses**

**Seroprevalence of SARS-CoV-2 antibodies and retrospective mortality in two African settings: Lubumbashi, Democratic Republic of Congo and Abidjan, Côte d’Ivoire**

Erica Simons^1$^, Birgit Nikolay^1*$^, Pascal Ouedraogo^1^_,_ Estelle Pasquier^1^, Carlos Tiemeni^3^, Ismael Adjaho^2^, Colette Badjo^2^, Kaouther Chamman^1^, Mariam Diomandé^2^, Mireille Dosso^4^, Moussa Doumbia^4^, Yves Asuni Izia^3^, Hugues Kakompe^5^, Anne Marie Katsomya^3^, Vicky Kij^5^, Viviane Kouakou Akissi^4^, Christopher Mambula^3^, Placide Mbala-Kingebenid^6^, Jacques Muzinga^7^, Basile Ngoy^5^, Klaudia Porten^1^_,_ Halidou Salou^1^, Daouda Sevede^4^, Francisco Luquero^1^, Etienne Gignoux^1^

***Affiliations***

^1^Epicentre, Paris, France

^2^Médecins Sans Frontières, Abidjan, Cote d’Ivoire

^3^Médecins Sans Frontières, Paris, France

^4^Institut Pasteur Cote d’Ivoire, Abidjan, Cote d’Ivoire

^5^Ministry of Health, Democratic Republic of Congo

^6^INRB, Democratic Republic of Congo

^7^Laboratoire National de Lubumbashi, Democratic Republic of Congo

birgit.nikolay@epicentre.msf.org

^$^These authors contributed equally to the study

1. **Study design and sampling methodology**

**Lubumbashi**

*Sample size*

The sample size for the mortality and seroprevalence survey was calculated using ENA software [https://smartmethodology.org/survey-planning-tools/smart-emergency-nutrition-assessment/]. For the mortality survey component, based on mortality estimates reported by World Population Prospects [https://population.un.org/wpp/] (crude death rate (CDR) of 0.25 per 10,000 persons per day in DRC), a precision of +/- 0.125, a recall period of at least 103 days (baseline: January 1, 2020-April 12 (Easter) 2020), a cluster effect of 1.5, and an average household size of 5.8 persons, a total of 1,545 households were to be enrolled in each stratum. We chose Easter as the start of the pandemic period as it was a time point easy to recall and sufficiently close to the detection of the first confirmed case.

For the seroprevalence component, to allow for estimates stratified by age (<20, 20-34, 35-49, 50+) and with statistical power to estimate expected seroprevalence with sufficient precision in the smallest age group, the sample size was based on those aged 50 years and older representing approximately 9.5% of the population. Assuming a prevalence of anti-SRAS-CoV-2 antibodies of 10% after the first wave (assumption before availability of estimates in the African context), a precision of ±5% (for each age group), 5% type 1 error, and 5% inconclusive results, at least 146 individuals per age group were to be included in the seroprevalence survey. To ensure that at least 146 individuals were enrolled in the 50+ age group, an overall sample size of 1532 individuals per stratum was required. Allowing for a ~15% margin for individuals who would refuse or be unable to participate in the seroprevalence study, a total of 309 households (1 household per cluster) were therefore required in each stratum for the seroprevalence study.

*Sampling methodology*

In the first stage, clusters were systematically sampled with a probability proportional to the respective population size of each neighborhood. Within each stratum, 309 points were randomly selected excluding non-residential areas. GPS coordinates for all initial points were downloaded GPS devices to physically locate the points. The first point represented the first household in a cluster. The remaining households were selected using a standard neighboring household selection process. In total, the goal was to include 5 households in the mortality survey and 1 household in the seroprevalence survey per cluster. The first household selected in each cluster was invited to participate in the seroprevalence survey. If a household declined to participate in either survey, the next household was invited to participate using the standard household selection process.

**Abidjan**

*Sample size*

Sample size calculations followed a similar rationale as for Lubumbashi.

For the mortality survey component, based on mortality estimates reported by World Population Prospects [https://population.un.org/wpp/] (crude mortality rate (CMR) of 0.3 per 10,000 persons per day in Côte d'Ivoire), a precision of +/- 0.15, a recall period of at least 117 days (first wave: April 13th – August 7th, 2020), a design effect of 1.5 and a household size of 5 people, a total of 1418 households were required for the mortality survey. We chose again Easter as the start of the pandemic period as it was a time point easy to recall and sufficiently close to the detection of the first confirmed case.

For the seroprevalence survey component, to allow for age-stratified (<20, 20-34, 35-49, 50+) estimates and assuming a SARS-CoV-2 antibody prevalence of 25% based on published studies in Africa, a precision of ±7%, 5% type 1 error and 5% inconclusive results, at least 154 individuals per age-group were required in the seroprevalence survey, resulting in an overall sample size of 1573 individuals per stratum. Allowing for a ~10% margin for individuals who would refuse or be unable to participate in the seroprevalence study, a total of 320 households (1 household per cluster) were therefore required in each stratum for the seroprevalence study.

*Sampling methodology*

In the first stage, clusters were systematically sampled with a probability of distribution proportional to the respective population size of each neighborhood (probability proportional to size). Subsequently, the points of clusters were selected using the Epicentre Geo-sampler tool (https://apps.msf.fr/epiGeoSampler/) to ensure selection of roof-tops and to exclude non-residential areas. GPS coordinates for all points were uploaded onto tablets and teams used the OsmAnd application to identify each GPS point, which represented the first household within a cluster. The remaining households were chosen following a standard selection process of nearby households. In total, the aim was to include 5 households in the mortality survey and 1 household in the seroprevalence survey per cluster with the first selected household in each cluster invited to participate in the seroprevalence survey. If a household was unavailable or refused participation in either survey, an additional household was asked to participate.

1. **Causes of death**

**Table A.** Principal causes of death, overall and by pre-pandemic/pandemic phase in Lubumbashi.

| **Cause of death** | **Pre-pandemic**  **(N=17)** | **Pandemic**  **(N=133)** | **Overall**  **(N=150)** |
| --- | --- | --- | --- |
| Isolated Fever/ Malaria | 17.6% (3) | 36.1% (48) | 34.0% (51) |
| Accident / Trauma / Violence | 17.6% (3) | 9.8% (13) | 10.7% (16) |
| Other respiratory diseases | 11.8% (2) | 6.0% (8) | 6.7% (10) |
| Cardiopathy/Arterial hypertension / Cancer/ Diabetes | 11.8% (2) | 6.0% (8) | 6.7% (10) |
| Pregnancy/ Birth/  Neonatal death | 0.0% (0) | 4.5% (6) | 4.0% (6) |
| Diarrhea | 0.0% (0) | 3.0% (4) | 2.7% (4) |
| Measles | 5.9% (1) | 0.0% (0) | 0.7% (1) |
| Malnutrition | 0.0% (0) | 0.8% (1) | 0.7% (1) |
| COVID-19 | 11.8% (2) | 0.0% (0) | 1.3% (2) |
| Other causes | 23.5% (4) | 21.8% (29) | 22.0% (33) |
| Unknown | 0.0% (0) | 12.0% (16) | 10.7% (16) |

**Table B.** Principal causes of death, overall and by pre-pandemic/pandemic phase in Abidjan.

| **Cause of death** | **Pre-pandemic**  **(N=27)** | **Pandemic**  **(N=56)** | **Overall**  **(N=83)** |
| --- | --- | --- | --- |
| Cardiopathy/Arterial hypertension / Cancer/ Diabetes | 40.7 (11) | 21.4 (12) | 27.7 (23) |
| Isolated Fever/ Malaria | 18.5 (5) | 25.0 (14) | 22.9 (19) |
| Other respiratory diseases | 11.1 (3) | 10.7 (6) | 10.8 (9) |
| Pregnancy/ Birth/  Neonatal death | 3.7 (1) | 1.8 (1) | 2.4 (2) |
| Accident / Trauma / Violence | 0.0 (0) | 5.4 (3) | 3.6 (3) |
| Other causes | 11.1 (3) | 12.5 (7) | 12.1 (10) |
| Unknown | 14.8 (4) | 23.2 (13) | 20.5 (17) |

1. **Mortality rates by age-groups**

**Table C.** Crude Mortality rates (/10 000 persons/day) by age-group and time period and rate ratio compared to pre-pandemic period in Lubumbashi.

| **Age group (years)** | **Pre-pandemic (95%CI)** | **Pandemic**  **(95%CI)** | **Rate ratio**  **(95%CI)** |
| --- | --- | --- | --- |
| < 5 ans | 0.04 (0.01-0.15) | 0.42 (0.30-0.60) | 11.9 (2.8-50.6)** |
| [5-20) | 0.08 (0.03-0.18) | 0.08 (0.05-0.13) | 1.1 (0.4-2.8) |
| [20-35) | 0.02 (0.01-0.09) | 0.07 (0.04-0.12) | 3.1 (0.7-14.1) |
| [35-50) | 0.10 (0.03-0.41) | 0.18 (0.11-0.29) | 1.7 (0.4-7.7) |
| 50+ | 0.58 (0.23-1.45) | 0.95 (0.69-1.31) | 1.6 (0.6-4.4) |
| ** p-value <0.05 | | | |

**Table D.** Crude Mortality rates (/10 000 persons/day) by age-group and time period and rate ratio compared to pre-pandemic period in Abidjan.

| **Age group (years)** | **Pre-pandemic (95%CI)** | **Pandemic**  **(95%CI)** | **Rate ratio**  **(95%CI)** |
| --- | --- | --- | --- |
| < 5 ans | 0.03 (0.01-0.15) | 0.11 (0.05-0.24) | 3.4 (0.6-18.3) |
| [5-20) | 0.02 (0.01-0.05) | 0.02 (0.01-0.06) | 1.2 (0.2-6.2) |
| [20-35) | 0.01 (0-0.06) | 0.03 (0.01-0.08) | 2.2 (0.4-11.3) |
| [35-50) | 0.03 (0.01-0.10) | 0.05 (0.02-0.12) | 1.8 (0.5-7.4) |
| 50+ | 0.26 (0.15-0.45) | 0.28 (0.18-0.42) | 1.1 (0.5-2.1) |

1. **Crude mortality rates (CMR) by SARS-CoV-2 waves**

**Table E.** CMR by SARS-CoV-2 waves in Lubumbashi.

| **Time period** | **N deaths** | **CMR per 10 000 per day (95%CI)** | **Rate ratio (95%CI)** | **p-value** |
| --- | --- | --- | --- | --- |
| Pre-pandemic  (01Jan20-12Apr20) | 17 | 0.08 (0.05-0.14) | Baseline | NA |
| Pandemic  (13Apr20-18May21) | 133 | 0.20 (0.17-0.25) | 2.5 (1.4-4.3) | p=0.001 |
| Wave 1  (13Apr20-31Aug20) | 30 | 0.12 (0.09-0.18) | 1.5 (0.8-2.9) | p=0.216 |
| Wave 2  (01Nov20-18May21) | 85 | 0.29 (0.23-0.37) | 3.5 (2.0-6.1) | p=<0.001 |

**Table F.** CMR by SARS-CoV-2 waves in Abidjan.

| **Time period** | **N deaths** | **CMR per 10 000 per day (95%CI)** | **Rate ratio (95%CI)** | **p-value** |
| --- | --- | --- | --- | --- |
| Pre-pandemic  (01Janv19-28Feb20) | 29 | 0.05 (0.03-0.07) | Baseline | NA |
| Pandemic  (13Apr20-10Nov21) | 54 | 0.07 (0.05-0.09) | 1.5 (0.9-2.5) | p=0.099 |
| Wave 1  (13Apr20-7Aug20) | 15 | 0.07 (0.04-0.13) | 1.6 (0.8-3.2) | p=0.215 |
| Wave 2  (01Jan21-30Jun21) | 15 | 0.06 (0.04-0.11) | 1.38 (0.7-2.8) | p=0.372 |
| Wave 3  (01July21-10Nov21) | 11 | 0.11 (0.06-0.2) | 2.38 (1.1-5.1) | p=0.024 |

1. **Sensitivity analysis – potential seasonal effects**

To account for potential seasonal effects, for Lubumbashi, we compared the baseline period (01 January-12^th^ April 2020) to the equivalent time period in 2021 (01 January-12 April 2020) (Table S7).

For Abidjan, we compared the third wave to its equivalent pre-pandemic period (Table S8). Moreover, we restricted the baseline period to 01 January-12 April 2020 (as for Lubumbashi) to test for any introduced bias due to the longer baseline period in Abidjan (Table S8).

The results are consistent with the main analysis.

**Table G.** Sensitivity analysis for seasonal effects, Lubumbashi.

| **Time period** | **N deaths** | **CMR per 10 000 per day (95%CI)** | **Rate ratio (95%CI)** | **p-value** |
| --- | --- | --- | --- | --- |
| **Equivalent time periods pre-pandemic & pandemic periods** | | | | |
| Pre-pandemic  (01Jan20-12Apr20) | 17 | 0.08 (0.05-0.14) | Baseline | NA |
| Pandemic equivalent  (01Jan21-12Apr21) | 53 | 0.32 (0.24-0.42) | 3.8 (2.1-6.9) | P<0.001 |

**Table H.** Sensitivity analysis for seasonal effects, Abidjan.

| **Time period** | **N deaths** | **CMR per 10 000 per day (95%CI)** | **Rate ratio (95%CI)** | **p-value** |
| --- | --- | --- | --- | --- |
| **Equivalent time periods pre-pandemic & wave 3** | | | | |
| Pre-pandemic equivalent  (01July19-10Nov19) | 7 | 0.04 (0.02-0.08) | Baseline | NA |
| Wave 3  (01July21-10Nov21) | 11 | 0.11 (0.06-0.20) | 3.0 (1.0-8.5) | p=0.043 |
| **Shorter pre-pandemic (as in Lubumbashi)** | | | | |
| Pre-pandemic short  (01Jan20-12Apr20) | 6 | 0.05 (0.02-0.13) | Baseline | NA |
| Pandemic  (13Apr20-10Nov21) | 54 | 0.07 (0.05-0.09) | 1.3 (0.5-3.2) | p=0.573 |

1. **Geographic differences in CMR**

**Table I.** CMR (/10 000 persons/day) by stratum in Lubumbashi.

| **Stratum** | **Pre-pandemic (95%CI)** | **Pandemic**  **(95%CI)** | **Rate ratio**  **(95%CI)** |
| --- | --- | --- | --- |
| Lubumbashi | 0.10 (0.05-0.20) | 0.1 (0.08-0.14) | 1.0 (0.5-2.1) |
| Kampemba/Tshamilemba | 0.07 (0.03-0.16) | 0.25 (0.20-0.31) | 3.4 (1.6-7.4) |

**Table J.** CMR (/10 000 persons/day) by stratum in Abidjan.

| **Stratum** | **Pre-pandemic (95%CI)** | **Pandemic**  **(95%CI)** | **Rate ratio**  **(95%CI)** |
| --- | --- | --- | --- |
| Marcory | 0.02 (0.01-0.04) | 0.04 (0.03-0.07) | 2.2 (0.9-5.2) |
| Yopougon | 0.05 (0.03-0.08) | 0.08 (0.05-0.11) | 1.5 (0.8-2.5) |

1. **Risk factors of household death**

**Table K.** Association of household death with socioeconomic risk factors in Lubumbashi.

| **Risk factor** |  | **OR (95%CI)** | **p-value** |
| --- | --- | --- | --- |
| House type | Individual | Reference |  |
|  | Semi-detached house | 1.5 (0.9-2.3) | 0.105 |
|  | Row house | 1.2 (0.8- 1.7) | 0.386 |
| Latrine availability | No | Reference |  |
|  | Yes | 1.4 (1.0-2.0) | 0.075 |
| Number people per room | ≤1 | Reference |  |
|  | >1-2 | 5.6 (2.4-13.2) | <0.001 |
|  | >2 | 8.1 (3.2-20.5) | <0.001 |

**Table L.** Association of household death with socioeconomic risk factors in Abidjan.

| **Risk factor** |  | **OR (95%CI)** | **p-value** |
| --- | --- | --- | --- |
| House type | Individual | Reference |  |
|  | House with common courtyard | 2.1 (1.2-3.8) | 0.014 |
|  | Semi-detached house | 2.6 (0.3-20.1) | 0.369 |
|  | Row house | 1.5 (0.6- 3.8) | 0.399 |
|  |  |  |  |
| Latrine availability | No | Reference |  |
|  | Yes | 1.0 (0.6-1.9) | 0.895 |
| Number people per room | ≤1 | Reference |  |
|  | >1-2 | 8.2 (1.1-61.1) | 0.041 |
|  | >2 | 7.6 (1.0-58.2) | 0.051 |

1. **Comparison of rapid diagnostic test (RDT) and ELISA/ECLIA results**

**Table M.** Comparison of RDT and ELISA results, Lubumbashi.

|  |  | ***ELISA*** | |
| --- | --- | --- | --- |
|  |  | *N pos ELISA /N tested* | *% pos ELISA* |
| *RDT* | ***Positive (IgG or IgM)*** | 273/302 | 90.4 |
|  | *IgG only* | 225/239 | 94.1 |
|  | *IgM only* | 19/33 | 57.6 |
|  | *IgG and IgM* | 29/30 | 96.7 |
|  | ***Negative*** | 563/1593 | 35.3 |

**Table N.** Comparison of RDT and ECLIA results, Abidjan (vaccinated and unvaccinated individuals).

|  |  | ***ECLIA*** | |
| --- | --- | --- | --- |
|  |  | *N pos ECLIA /N tested* | *% pos ECLIA* |
| *RDT* | ***Positive (IgG or IgM)*** | 749/769 | 97.4 |
|  | *IgG only* | 657/672 | 97.8 |
|  | *IgM only* | 41/46 | 89.1 |
|  | *IgG and IgM* | 51/51 | 100.0 |
|  | ***Negative*** | 760/1020 | 74.5 |

1. **Geographic differences in seroprevalence**

**Table O.** Seroprevalence by stratum in Lubumbashi.

| **Stratum** | **RDT (95%CI)** | **ELISA (95%CI)** |
| --- | --- | --- |
| Lubumbashi | 12.4 (9.7-15.4) | 43.8 (39.2-48.5) |
| Kampemba/Tshamilemba | 17.3 (14.4-20.4) | 42.9 (38.8-47.1) |

**Table P.** Seroprevalence by stratum in Abidjan.

| **Stratum** | **RDT (95%CI)** | **ECLIA (95%CI)** |
| --- | --- | --- |
| **Phase 1 :** |  |  |
| Marcory | 13.1 (9.0-18.1) | 77.2 (71.1-82.5) |
| Yopougon | 18.4 (13.5-24.1) | 71.9 (65.3-78.0) |
| **Phase 2 :** |  |  |
| Marcory | 44.3 (37.0-51.8) | 90.0 (85.2-93.7) |
| Yopougon | 37.9 (32.6-43.5) | 80.9 (76.1-85.3) |

**Table Q.** Investigated risk factors for seropositivity based on RDT, Lubumbashi.

|  | **OR (95%CI)** | **p-value** |
| --- | --- | --- |
| **Working** |  |  |
| No | Baseline |  |
| Yes | 0.79 (0.46-1.36) | 0.396 |
| **Work commute** |  |  |
| Individual | Baseline |  |
| Shared | 1.37 (0.50-3.73) | 0.543 |
| **Commute frequency** |  |  |
| Not every day | Baseline |  |
| Nearly every day | 0.33 (0.12-0.93) | 0.037 |
| **Eating in restaurant** |  |  |
| No | Baseline |  |
| Yes | 0.53 (0.19-1.54) | 0.246 |
| **Number of contacts per day (closed settings)** |  |  |
| <5 | Baseline |  |
| 5-<10 | 2.91 (0.76-11.08) | 0.120 |
| 10-20 | 3.86 (0.94-15.79) | 0.062 |
| >=20 | 1.11 (0.19-6.71) | 0.910 |
| **Number of contacts per day (open settings)** |  |  |
| <5 | Baseline |  |
| 5-<10 | 0.51 (0.16-1.67) | 0.269 |
| 10-20 | 0.27 (0.07-1.01) | 0.053 |
| >=20 | 0.78 (0.20- 2.96) | 0.714 |

**Table R.** Investigated risk factors for seropositivity among non-vaccinated individuals based on RDT, Abidjan.

|  | **OR (95%CI)** | **p-value** |
| --- | --- | --- |
| **Working** |  |  |
| No | Baseline |  |
| Yes | 0.85 (0.57-1.25) | 0.402 |
| **Work commute** |  |  |
| Individual | Baseline |  |
| Shared | 1.45 (0.78-2.69) | 0.245 |
| **Commute frequency** |  |  |
| Not every day | Baseline |  |
| Nearly every day | 0.88 (0.42-1.84) | 0.734 |
| **Eating in restaurant** |  |  |
| No | Baseline |  |
| Yes | 0.99 (0.62-1.59) | 0.978 |
| **Number of contacts per day (closed settings)** |  |  |
| <5 | Baseline |  |
| 5-<10 | 0.92 (0.58-1.45) | 0.714 |
| 10-20 | 0.98 (0.56-1.70) | 0.938 |
| >=20 | 0.61 (0.33-1.15) | 0.126 |
| **Number of contacts per day (open settings)** |  |  |
| <5 | Baseline |  |
| 5-<10 | 1.24 (0.68-2.24) | 0.482 |
| 10-20 | 0.92 (0.50-1.70) | 0.798 |
| >=20 | 0.88 (0.48 – 1.64) | 0.697 |
