# Supplementary material for: Seroprevalence of SARS-CoV-2 antibodies and retrospective mortality in two African settings: Lubumbashi, Democratic Republic of the Congo and Abidjan, Côte d’Ivoire
Source: PLOS Glob Public Health. 2023 Jun 8;3(6):e0001457. doi: 10.1371/journal.pgph.0001457 (PMC10249894; doi:10.1371/journal.pgph.0001457)
Supplement: S2 Text — (DOCX) [file pgph.0001457.s002.docx]

###

### **S2 Text**

### **GENERIC QUESTIONNAIRES**

**Seroprevalence of SARS-CoV-2 antibodies and retrospective mortality in two African settings: Lubumbashi, Democratic Republic of Congo and Abidjan, Côte d’Ivoire**

Erica Simons^1$^, Birgit Nikolay^1*$^, Pascal Ouedraogo^1^_,_ Estelle Pasquier^1^, Carlos Tiemeni^3^, Ismael Adjaho^2^, Colette Badjo^2^, Kaouther Chamman^1^, Mariam Diomandé^2^, Mireille Dosso^4^, Moussa Doumbia^4^, Yves Asuni Izia^3^, Hugues Kakompe^5^, Anne Marie Katsomya^3^, Vicky Kij^5^, Viviane Kouakou Akissi^4^, Christopher Mambula^3^, Placide Mbala-Kingebenid^6^, Jacques Muzinga^7^, Basile Ngoy^5^, Klaudia Porten^1^_,_ Halidou Salou^1^, Daouda Sevede^4^, Francisco Luquero^1^, Etienne Gignoux^1^

***Affiliations***

^1^Epicentre, Paris, France

^2^Médecins Sans Frontières, Abidjan, Cote d’Ivoire

^3^Médecins Sans Frontières, Paris, France

^4^Institut Pasteur Cote d’Ivoire, Abidjan, Cote d’Ivoire

^5^Ministry of Health, Democratic Republic of Congo

^6^INRB, Democratic Republic of Congo

^7^Laboratoire National de Lubumbashi, Democratic Republic of Congo

[birgit.nikolay@epicentre.msf.org](mailto:birgit.nikolay@epicentre.msf.org)

^$^These authors contributed equally to the study

**Section 1: Mortality Questionnaire**

**Team:** ___|___| **Interview Date:** |___|___|2020 **Cluster Nb:** |___|___|___| **Household Number:** |___|___|

**Consent** (**0**: No, **1**: Yes) |___| **GPS ID:** |_____| **Selected for seroprevalence survey**: (**0**: No, **1**: Yes) |___|

**Type of household**: |___| (list) **Number of rooms:** |___| **Number of bedrooms:** |___| **Is the latrine/toilet shared with other HHs?:** |___|

**Complete one line for each member of the household since January 1^st^ 2020**

| **N** | **Rapid test** | **Sex** | **Age** | | | **Arrived during recall period** | **Left during recall period** | **Born during recall period** | **Died during recall period** | **For each death in the cluster, complete further details on the following form (one line per death)** |
| --- | --- | --- | --- | --- | --- | --- | --- | --- | --- | --- |
|  | 1=Yes  0=No | 1=M  2=F | Years | Months | Days | Date of arrival (dd/mm/yyyy | Date of departure (dd/mm/yyyy) | Date of birth  (dd/mm/yyyy) | Date of death  (dd/mm/yyyy) |  |
| **1** |  |  |  |  |  |  |  |  |  |  |
| **2** |  |  |  |  |  |  |  |  |  |  |
| **3** |  |  |  |  |  |  |  |  |  |  |
| **4** |  |  |  |  |  |  |  |  |  |  |
| **5** |  |  |  |  |  |  |  |  |  |  |
| **6** |  |  |  |  |  |  |  |  |  |  |
| **7** |  |  |  |  |  |  |  |  |  |  |
| **8** |  |  |  |  |  |  |  |  |  |  |
| **9** |  |  |  |  |  |  |  |  |  |  |
| **10** |  |  |  |  |  |  |  |  |  |  |

**Has anyone one in the household current symptoms of COVID-19*?** (**0**: No, **1**: Yes, **9** Don’t know) |___| If yes, specify the household member ID number(s) |______________|

*****A COVID-19 suspected case is someone with acute onset of fever and cough or any three or more of the following sings/symptoms: sore throat, coryza, general weakness/fatigue, headache, cough, runny nose, shortness of breath, anorexia/nausea/vomiting, diarrhoea, altered mental status, myalgia. Suspected cases should be referred for PCR testing

**Type of Household:** 1) Single detached house 2) Semi-detached house 3) Row house 4) Apartment or flat in a duplex 5) Apartment in a building that has five or more storeys

6) Apartment in a building with fewer than five storeys 7) Other single-attached house (i.e. Housing attached to non-residential structure, like store) 8) Moveable dwelling (e.g. mobile home)

**Team:** |___|___| **Interview Date:** |___|___|2020 **Cluster Nb:** |___|___|___|

**For the entire cluster, complete one line for each household member who has died since January 1^st^, 2020**

| No | HH Nb | HH member number  ["N" on the 1st question page]. | Place of death | Death certif./ medical report with cause of death | Cause of death | Do you think he/she died of COVID? | Medical History/Co-morbidities | Unexpected or rapid death? | Duration of illness /injuries before death? | Any of the following symptoms prior to death? | Contact *(within 1 month****)*** with person with positive COVID test or symptoms? | Sought care within 1 month prior to death? | **If yes**, which type? | Has he/she received a COVID test? | If care not sought, why not? |
| --- | --- | --- | --- | --- | --- | --- | --- | --- | --- | --- | --- | --- | --- | --- | --- |
|  |  |  | **List 1** | =No 1=Yes 99=DNK | **List 2** | 0=No 1=Yes 99=DNK | **List 3** Multiple responses possible | 0=No 1=Yes 99=DNK | Nb. days **Write DNK if unknown** | **List 4** Multiple responses possible | 0=No 1=Yes 99=DNK | 0=No 1=Yes 99=DNK | **List 5** Multiple responses possible | **List 6** | **List 7** Multiple responses possible |
| 1 |  |  |  |  |  |  |  |  |  |  |  |  |  |  |  |
| 2 |  |  |  |  |  |  |  |  |  |  |  |  |  |  |  |
| 3 |  |  |  |  |  |  |  |  |  |  |  |  |  |  |  |
| 4 |  |  |  |  |  |  |  |  |  |  |  |  |  |  |  |
| 5 |  |  |  |  |  |  |  |  |  |  |  |  |  |  |  |
| 6 |  |  |  |  |  |  |  |  |  |  |  |  |  |  |  |
| 7 |  |  |  |  |  |  |  |  |  |  |  |  |  |  |  |
| 8 |  |  |  |  |  |  |  |  |  |  |  |  |  |  |  |
| 9 |  |  |  |  |  |  |  |  |  |  |  |  |  |  |  |
| 10 |  |  |  |  |  |  |  |  |  |  |  |  |  |  |  |

List of codes

**Questions related to funeral practices:**

Is the grave identified with the name of the deceased? (**0**: No, **1**: Yes, **9** Don’t know) |___|

How many days do you keep the body before burial (on average)? |___|

What funeral practices do you carry out? |___| (List 8)

Where do you usually bury the deceased? |___| (List 9)

On what type of land do you bury the deceased? |___| (List 10)

List of codes

| **List 1 – Place of death** | | | |
| --- | --- | --- | --- |
| **1** | | | Home |
| **2** | | | On the way to the health centre/hospital |
| **3** | | | Hospital (Specify) |
| **4** | | | Health Centre (Specify) |
| **5** | | | Traditional practitioner |
| **6** | | | Other (Specify) |
| **99** | | | Don't know |
|  | | | |
| **List 2 – Cause of death** | | | |
| **1** | COVID-19 | | |
| **2** | Isolated fever / Malaria | | |
| **3** | Diarrhoea | | |
| **4** | (Other) Respiratory diseases | | |
| **5** | Malnutrition | | |
| **6** | Measles | | |
| **7** | Neonatal death/disease | | |
| **8** | Death/disease during pregnancy | | |
| **9** | Death/disease during childbirth | | |
| **10** | Death/disease after delivery (0 - 42 d) | | |
| **11** | Accident / Trauma | | |
| **12** | Violence | | |
| **13** | Other (Specify) | | |
| **99** | Don't know | | |
|  | | | |
| **List 3 – COVID symptoms** | | | |
| **1** | | | Fever |
| **2** | | | Cough |
| **3** | | | Shortness of breath |
| **4** | | | Weakness/fatigue |
| **5** | | | Extreme fatigue* |
| **6** | | | Headaches |
| **7** | | | Muscle pain |
| **8** | | | Sore throat |
| **9** | | | Runny nose |
| **10** | | | Loss of appetite/vomiting/nausea |
| **11** | | | Diarrhoea |
| **12** | | | Change in mental state |
| **13** | | | Loss of taste/odour |
| **0** | | | No symptoms |
| **99** | | | Don't know |
|  | | | |
| **List 4 – Medical history/comorbidities** | | | |
| **1** | | | Hypertension |
| **2** | | | Coronary Heart Disease |
| **3** | | | Congestive Heart Failure |
| **4** | | | Asthma |
| **5** | | | Chronic lung disease |
| **6** | | | HIV |
| **7** | | | Kidney Disease |
| **8** | | | Cirrhosis |
| **9** | | | Hep B |
| **10** | | | Hep C |
| **10** | | | Obesity (BMI >35) |
| **11** | | | Diabetes |
| **12** | | | Cancer |
| **13** | | | Past transplant (any organ) |
| **14** | | | Autoimmune disease (Polyarthritis, Crohn, Lupus, Multiple Sclerosis…) |
| **15** | | | Current smoker |
|  | | | |
| **List 5 – Type of care** | | | |
| **1** | | Self-medication: modern medicine | |
| **2** | | Self-medication: traditional medicine | |
| **3** | | Buying drugs at the market | |
| **4** | | Traditional practitioner | |
| **5** | | Pharmacist | |
| **6** | | Health Centre (specify) | |
| **7** | | Hospital (specify) | |
| **8** | | Other (specify) | |
| **99** | | Don't know | |
|  | | | |
| **List 6 – COVID test** | | | |
| **0** | | | No, no COVID test |
| **1** | | | Yes, positive |
| **2** | | | Yes, negative |
| **3** | | | Yes, do not know the result |
| **99** | | | Don't know |
|  | | | |
| **List 7 – Reasons for not receiving care in a health care facility** | | | |
| **1** | | | Rapid/immediate death |
| **2** | | | No money/care too expensive |
| **3** | | | No money/ transport too expensive |
| **4** | | | Too sick to seek care |
| **5** | | | Not sick enough to seek care |
| **6** | | | Did not have the time/too busy |
| **7** | | | Purchased products at the market/pharmacy |
| **8** | | | Received traditional care |
| **9** | | | Health centre too far away |
| **10** | | | Health centre closed and/or no HWs there |
| **11** | | | No medication at the health centre |
| **12** | | | Overcrowded health centre |
| **13** | | | Poor quality health centre |
| **14** | | | Refused care at the health centre |
| **15** | | | No confidence in health services |
| **16** | | | Confinement (COVID-19) |
| **17** | | | Fear of COVID-19 |
| **18** | | | Other (specify) |
| **99** | | | Don't know |

| **List 8 – Type of funeral practice** | | |
| --- | --- | --- |
| **1** | | Burial alone in a grave |
| **2** | | Burial of several deceased in the same grave |
| **3** | | Cremation |
| **4** | | Other (Specify) |
| **99** | | Do not know |
|  | | |
| **List 9 – Location of burial** | | |
| **1** | In Lubumbashi town | |
| **2** | In the surroundings of the town | |
| **3** | In the family field/village of origin | |
| **4** | Other (Specify) | |
| **99** | Do not know | |
|  | | |
| **List 10– Type of land for burial** | | |
| **1** | | Official Cemetery |
| **2** | | Private land |
| **3** | | Unofficial "Cemetery" (place where there are a large number of graves) |
| **4** | | Wasteland |
| **5** | | In the family plot |
| **6** | | Other (Specify) |
| **99** | | Do not know |

| **Section 2: Seroprevalence (for all participants who are eligible and have consented to serology testing)** | | | | | |
| --- | --- | --- | --- | --- | --- |
| **Surveyor Info** | **Surveyor’s initial** | | | **Date of interview (DD/MM/YYYY) ___/___/___ Inclusion site List of codes** | |
| **Cluster ID:** | **Household ID:** | | | **Household Member Nb:** | |
| **Results of the Biosynex COVID-19 BSS rapid test:** | | | | | |
| □ Positive- IgG □ Positive- IgM s □ Positive- IgG and IgM □ Negative □ Invalid | | | | | |
| **COVID-19 Symptoms** | | | | | |
| Since January, have you had any of the following: | | | | | |
| Fever ≥38°C | | □ Yes □ No | Chest pain | | □ Yes □ No |
| Chills | | □ Yes □ No | Other respiratory symptoms | | □ Yes □ No |
| Fatigue | | □ Yes □ No | Headache | | □ Yes □ No |
| Muscle ache (myalgia) | | □ Yes □ No | Nausea/vomiting | | □ Yes □ No |
| Sore throat | | □ Yes □ No | Abdominal pain | | □ Yes □ No |
| Cough | | □ Yes □ No | Diarrhoea | | □ Yes □ No |
| Runny nose (rhinorrea) | | □ Yes □ No | Loss of taste | | □ Yes □ No |
| Shortness of breath | | □ Yes □ No | Loss of smell | | □ Yes □ No |
| Wheezing | | □ Yes □ No | Altered mental status | | □ Yes □ No |
| If 1 or more signs, when did you experienced them (approx.)? *[adapt dates based on recall period and date of survey]* | | | □ 01-15 Jan □ 16-31 Jan □ 01-15 Feb □ 16-28 Feb □ 01-15 Mar □ 16-30 Mar □ 01-15 April  □ 16-31 April □ 01-15 May □ 16-31 May □ 01-15 June □ 16-30 June □ 01-15 July □ 16-31 July  □ 01-15 August □ 16-31 August □ 01-15 September □ 16-31 September □ Currently* □ DNK | | |
| How long did they last (approx.)? | | | □ < 2 days □ 2 days to 1 week □ 1 to 2 weeks □ 2 to 3 weeks □ > 3 weeks □ DNK | | |
| Did you see a doctor for any of these signs? | | | □ Yes □ No □ DNK | | |
| Did you receive a COVID-19 test? | | | □ Yes □ No □ DNK | | |
| If yes, when (approx.)? *[adapt dates based on recall period and date of survey]* | | | □ 01-15 Jan □ 16-31 Jan □ 01-15 Feb □ 16-28 Feb □ 01-15 Mar □ 16-30 Mar □ 01-15 April  □ 16-31 April □ 01-15 May □ 16-31 May □ 01-15 June □ 16-30 June □ 01-15 July □ 16-31 July  □ 01-15 August □ 16-31 August □ 01-15 September □ 16-31 September □ DNK | | |
| What was the final result of the COVID test? | | | □ Positive □ Negative □ Undetermined □ Never received the results □ DNK | | |
| Did you go the hospital?  Were you hospitalized?  If yes, when and where?  Were you admitted to an intensive care unit during your hospital stay? | | | □ Yes □ No □ DNK  □ Yes □ No □ DNK  Where:_______________________________When:____________________________________  □ Yes □ No □ DNK | | |

| **For adults only- Occupational and social exposure** | | | | | | | | | |
| --- | --- | --- | --- | --- | --- | --- | --- | --- | --- |
| Do you work? | | | | | | □ Yes □No □ not specified/not answered | | | |
| Are you a healthcare worker? | | | | | | □ Yes □No □ not specified/not answered | | | |
| How often do you go to work? | | | | | | □ Almost every day □ Sometimes □ Rarely □ Never | | | |
| Which type of transport do you use to go to work?  If other, specify | | | | | | □Walk □Bike □ Private car □ Taxi □ Bus □ Other  ___________________________ | | | |
| Where do you spend most of the time during your work duties? | | | | | | □Indoors □Outdoors □Both indoors and outdoors | | | |
| Do you attend any social gatherings? | | | | | | □ Almost every day □ Sometimes □ Rarely □ Never | | | |
| On average, how many times per week do you eat in restaurants/food service outside of your home? | | | | | | _______ number of times | | | |
| On average, every day: how many people are you in contact with (distance <1m for >15min) inside your home? | | | | | | □ Nobody □ 1 person □ 2 to 4 □ 5 to 10 □ 10 to 20 □ > 20 | | | |
| On average, every day : how many people are you in contact with (distance <1m for >15min) outside of your home ? | | | | | | □ Nobody □ 1 person □ 2 to 4 □ 5 to 10 □ 10 to 20 □ > 20 | | | |
| **COVID-19 protection measures** | | | | | | | | | |
| Which measure(s) do you actually follow to protect yourself against a COVID-19 infection : | | | | | | | | | |
| Regular handwashing (soap, gel…) | | □ Yes □ No □ Unknown | Sneezing/coughing into my elbow | | | | | □ Yes □ No □ Unknown | |
| Wearing a face mask (any type) and if not why? | | □ Yes □ No □ Unknown  If no, why not? (List) | Disinfecting my everyday tools | | | | | □ Yes □ No □ Unknown | |
| Avoiding direct physical contact (handshake, greetings…) | | □ Yes □ No □ Unknown | Disinfecting my place of living | | | | | □ Yes □ No □ Unknown | |
| If you had symptoms of COVID-19, what would you do? (multiple responses possible) | | | | □ Go to clinic □ Call toll free number □ Go for COVID-19 test □ Stay at home  □ Stop attending social gatherings □ Keep a distance of at least 2 meters □ Inform people of illness/symptoms □ Wash hands more frequently | | | | |  |
| Who is at higher risk of getting severely sick from COVID? | | | | □ Everyone □ Elderly □ Children □ Pregnant Women □ People with already weak/compromised immune systems □ Diabetics □ People with cardiovascular problems | | | | |  |
| **Medical History/Comorbidities** | | | | | | | | | |
| Hypertension | □ Yes □ No □ Unknown | | Hep B | | | | | □ Yes □ No □ Unknown | |
| Coronary Heart Disease | □ Yes □ No □ Unknown | | Hep C | | | | | □ Yes □ No □ Unknown | |
| Congestive Heart Failure | □ Yes □ No □ Unknown | | Diabetes | | | | | □ Yes □ No □ Unknown | |
| Asthma | □ Yes □ No □ Unknown | | Cancer | | | | | □ Yes □ No □ Unknown | |
| Chronic cough or difficulty breathing | □ Yes □ No □ Unknown | | Past transplant (any organ) | | | | | □ Yes □ No □ Unknown | |
| HIV | □ Yes □ No □ Unknown | | Autoimmune disease (Polyarthritis, Crohn, Lupus, MS…) | | | | | □ Yes □ No □ Unknown | |
| Kidney Disease | □ Yes □ No □ Unknown | | Smoking habits | | | | | □ Current Smoker □ Former Smoker □ Never smoked | |
| Cirrhosis | □ Yes □ No □ Unknown | |  | | | | |  | |
| **Ongoing Treatments** | | | | | | | | | |
| Corticosteroids | | | □ Yes □ No □ DNK | | Cancer treatment | | □ Yes □ No □ DNK | | |
| Cyclophosphamide/cyclosporines(Antibiotics?) | | | □ Yes □ No □ DNK | | Post-Transplant treatment | | □ Yes □ No □ DNK | | |
| Monoclonal antibodies | | | □ Yes □ No □ DNK | |  | |  | | |

* Those currently fulfilling the COVID-19 suspected case definition should be referred for PCR testing. A COVID-19 suspected case is someone with acute onset of fever and cough or any three or more of the following sings/symptoms: sore throat, coryza, general weakness/fatigue, headache, cough, runny nose, shortness of breath, anorexia/nausea/vomiting, diarrhoea, altered mental status, myalgia. Suspected cases should be referred for PCR test
