# Supplementary material for: Seroprevalence of SARS-CoV-2 antibodies and retrospective mortality in two African settings: Lubumbashi, Democratic Republic of the Congo and Abidjan, Côte d’Ivoire
Source: PLOS Glob Public Health. 2023 Jun 8;3(6):e0001457. doi: 10.1371/journal.pgph.0001457 (PMC10249894; doi:10.1371/journal.pgph.0001457)
Supplement: Attachment — Submitted filename: Response_to_reviewers.docx [file pgph.0001457.s003.docx]

PGPH-D-22-01970
Seroprevalence of SARS-CoV-2 antibodies and retrospective mortality in two African settings: Lubumbashi, Democratic Republic of the Congo and Abidjan, Côte d’Ivoire
PLOS Global Public Health

Dear Dr. Nikolay,

Thank you for submitting your manuscript to PLOS Global Public Health. After careful consideration, we feel that it has merit but does not fully meet PLOS Global Public Health’s publication criteria as it currently stands. Therefore, we invite you to submit a revised version of the manuscript that addresses the points raised during the review process.

***Authors’ response:*** *We thank the Editor and Reviewer for their comments and provide responses below and in the manuscript with tracked changes.*

1) Journal Requirements:

In the online submission form, you indicated that "The minimal dataset underlying the findings of this study is available on request, in accordance with the legal framework set forth by Médecins Sans Frontières (MSF) data sharing policy (Karunakara U, PLoS Med 2013). MSF is committed to share and disseminate health data from its programs and research in an open, timely, and transparent manner in order to promote health benefits for populations while respecting ethical and legal obligations towards patients, research participants, and their communities. The MSF data sharing policy ensures that data will be available upon request to interested researchers while addressing all security, legal, and ethical concerns. All readers may contact [data.sharing@msf.org](mailto:data.sharing@msf.org) or Nouha TOUATI ([nouha.touati@epicentre.msf.org](mailto:nouha.touati@epicentre.msf.org)) to request data.. All PLOS journals now require all data underlying the findings described in their manuscript to be freely available to other researchers, either 1. In a public repository, 2. Within the manuscript itself, or 3. Uploaded as supplementary information.
This policy applies to all data except where public deposition would breach compliance with the protocol approved by your research ethics board. If your data cannot be made publicly available for ethical or legal reasons (e.g., public availability would compromise patient privacy), please explain your reasons by return email and your exemption request will be escalated to the editor for approval. Your exemption request will be handled independently and will not hold up the peer review process, but will need to be resolved should your manuscript be accepted for publication. One of the Editorial team will then be in touch if there are any issues.

***Authors’ response:*** *(We also replied directly to the email as requested)*

*As MSF is subject to the European Union General Data Protection Regulation (GDPR), the data collected in the framework of a study it carries out, and moreover as a sponsor, are also subject to the GDPR which ensures them a certain protection. The study data contains sensitive and potentially identifying information and cannot be sufficiently anonymized to meet GDPR standards and still retain their utility. Making them publicly available would compromise participant privacy. Thus, pseudonymized data (i.e. minimal dataset underlying the findings of this study) will be made available upon request and execution of a data sharing agreement or alternate means that allows assurance that principles of GDPR regulations will be met.*

Please review your reference list to ensure that it is complete and correct. If you have cited papers that have been retracted, please include the rationale for doing so in the manuscript text, or remove these references and replace them with relevant current references. Any changes to the reference list should be mentioned in the rebuttal letter that accompanies your revised manuscript. If you need to cite a retracted article, indicate the article’s retracted status in the References list and also include a citation and full reference for the retraction notice.

**Authors’ response:** We thank the editor for this comment. We have reviewed the reference list, assured that it is in the Vancouver style and can confirm that there are no retracted articles referenced in the manuscript text. Two added references were included as a response to the reviewers comments regarding performance of Biosynex RDT, Euroimmun ELISA, and Roche ECLIA (25-26).

**Reviewers' comments:**

**Reviewer #1:**

This paper presents the results of a retrospective mortality survey and cross-sectional survey in two locations in Africa – one in the DRC and one in Cote D’Ivoire. The finding of increased mortality during the pandemic among <5 year-olds is particularly striking and a timely reminder of the importance of maintaining access to healthcare in these settings. I think this is a worthwhile addition to the literature and I have minor comments only.

***Authors’ response:*** *We thank the reviewer for the positive assessment of our study.*

Minor comments:
Line 113: Why was January 1 2020 chosen for Lubumbashi and not January 1 2019? Could you perform a sensitivity analysis limiting the recall period to 1 January 2020 in Abidjan, to assess the possibility of bias due to the different recall periods and seasonality?

***Authors’ response:*** *We thank the reviewer for this pertinent question. January 1^st^, 2020 was chosen for Lubumbashi to limit recall bias and after review of official integrated disease surveillance data that did not show evidence of seasonal effects we decided that a longer baseline period was not necessary. The Lubumbashi protocol was developed independently of the Abidjan study where no such data was available to be examined, and where we therefore opted for a longer baseline period to be able to account for potential seasonality. We performed several sensitivity analyses to investigate for potential seasonal effects. For Lubumbashi, we compared the pre-pandemic period (01 January- 12 April 2020) to the exact same time period in 2021 (01 January- 12 April 2021). For Abidjan, we compared the period of the 3^rd^ wave (01 July 2021- 10 November 2021), where an increase was detected, to the same time period in the pre-pandemic period (01 July 2019- 10 November 2019). Following the reviewer suggestions we now also include a sensitivity analysis shortening the pre-pandemic period for Abidjan to 01 January 2020-12 April 2020.*

*All sensitivity analyses showed consistent results with the main findings.*

*We now mention the sensitivity analysis in the main manuscript:*

*Line 212 (clean version): We performed a sensitivity analysis to investigate if observed increases during the pandemic period in Lubumbashi and the 3rd wave in Abidjan may have been caused by seasonal effects. The results of the sensitivity analysis showed consistent results with the main analysis (Supplementary tables 7 and 8).*

*And we specified in the limitations section:*

*Line 315: Moreover, we were not able to adjust for potential remaining confounding such as seasonality of other circulating pathogens. A sensitivity analyses restricting the analysis to the same months during the pre-pandemic and the pandemic phase showed however consistent results.*

*And provide the details of the sensitivity analysis in the Supplementary material:*

1. ***Sensitivity analysis – potential seasonal effects***

*To account for potential seasonal effects, for Lubumbashi, we compared the baseline period (01 January-12^th^ April 2020) to the equivalent time period in 2021 (01 January-12 April 2020) (Table S7).*

*For Abidjan, we compared the third wave to its equivalent pre-pandemic period (Table S8). Moreover, we restricted the baseline period to 01 January-12 April 2020 (as for Lubumbashi) to test for any introduced bias due to the longer baseline period in Abidjan (Table S8).*

*The results are consistent with the main analysis.*

***Table S7.*** *Sensitivity analysis for seasonal effects, Lubumbashi.*

| ***Time period*** | ***N deaths*** | ***CMR per 10 000 per day (95%CI)*** | ***Rate ratio (95%CI)*** | ***p-value*** |
| --- | --- | --- | --- | --- |
| ***Equivalent time periods pre-pandemic & pandemic periods*** | | | | |
| *Pre-pandemic*  *(01Jan20-12Apr20)* | *17* | *0.08 (0.05-0.14)* | *Baseline* | *NA* |
| *Pandemic equivalent*  *(01Jan21-12Apr21)* | *53* | *0.32 (0.24-0.42)* | *3.8 (2.1-6.9)* | *P<0.001* |

***Table S8.*** *Sensitivity analysis for seasonal effects, Abidjan.*

| ***Time period*** | ***N deaths*** | ***CMR per 10 000 per day (95%CI)*** | ***Rate ratio (95%CI)*** | ***p-value*** |
| --- | --- | --- | --- | --- |
| ***Equivalent time periods pre-pandemic & wave 3*** | | | | |
| *Pre-pandemic equivalent*  *(01July19-10Nov19)* | *7* | *0.04 (0.02-0.08)* | *Baseline* | *NA* |
| *Wave 3*  *(01July21-10Nov21)* | *11* | *0.11 (0.06-0.20)* | *3.0 (1.0-8.5)* | *p=0.043* |
| ***Shorter pre-pandemic (as in Lubumbashi)*** | | | | |
| *Pre-pandemic short*  *(01Jan20-12Apr20)* | *6* | *0.05 (0.02-0.13)* | *Baseline* | *NA* |
| *Pandemic*  *(13Apr20-10Nov21)* | *54* | *0.07 (0.05-0.09)* | *1.3 (0.5-3.2)* | *p=0.573* |

Line 117: I’m unclear on how the survey questions were worded here. Were household members asked how many deaths occurred in each of these periods of time (including the COVID-19 waves)? Or were they asked the exact date of death, and only asked about the periods if they could not recall the exact date of death? The wording “12 April 2020 was chosen as cut-off date between the pre-pandemic and pandemic period as it was the Easter Sunday, a date easy to recall for survey participants.” makes it seem like the former (i.e. exact date not ascertained), but this justification doesn’t make sense given the detail that is required in lines 118-120. Please clarify how the information on date of death was obtained.

***Authors’ response:*** *We collected exact dates of deaths as part of the survey questions. We then used these dates to attribute the deaths to pre-pandemic and post-pandemic periods, as well as to the COVID waves, for the purpose of the analysis. We clarified this in the methods section as follows:
Line 89: “The mortality questionnaire consisted of sections covering housing characteristics, demographic data, and information on deaths (including the date of death) that occurred during the recall period.”*

*Line 123: “Deaths were attributed to the time periods based on their date of death. “*

Line 162 and 169: what does “matched” mean in this context?

***Authors’ response:*** *We intended to obtain RDT and ELISA/ECLIA results from all study participants, however in a few cases only a single test result was obtained. For the comparison of test results we could only use the data of individuals that had both test results. We revised the phrases to clarify:*

*Line 164: “Both diagnostic type results, RDT and ELISA, were available for 1,897 individuals.”*

*Line 172: “Both diagnostic type results, RDT and ECLIA, were available for 1,800 individuals.”*

Line 188-193: does “reported” here mean self-reported by household members?

***Authors’ response:*** *Indeed, these are deaths reported by the household heads. We modified the parts in the text to clarify:*

*Line 186: “In Lubumbashi, 150 deaths were reported by household heads during the entire recall period (1 January 2020 – 18 May 2021), including 17 deaths during the pre-pandemic and 133 deaths during the pandemic period (Figure 2A).* *In Abidjan, 83 deaths were reported by household heads during the recall period (1 January 2019 – 10 November 2021), including 29 deaths during the pre-pandemic and 54 during the pandemic period (Figure 2B). ”*

Line 307-311: is there any data on the sensitivity of the RDT and EUROIMMUN over time since infection/symptom onset (i.e. the proportion of individuals who serorevert), that you could report here? Given the high se/sp reported in the methods for the RDT, and the results you have found, I suspect the RDT wanes quickly and therefore a positive RDT could be an indication of a “recent” infection. Therefore, it might be worth mentioning this interpretation, e.g. in your interpretation of the shape of the RDT seropositivity by age curve and the differences between Phase 1 and Phase 2 in Abidjan.

***Authors’ response:*** *We thank the reviewer for the suggestion. We now have included additional citations for available studies that have examined the performance of Biosynex (the RDT used in both sites), Euroimmun (the ELISA used in Lubumbashi), and Roche (the ECLIA used in Abidjan).*

*Line 322: One study observed 91.3% and 83.7% of COVID-19 positive healthcare workers tested positive for IgM and IgG, respectively, with Biosynex one month after infection versus 51.8% IgM and 56.8% IgG at months 11-13 (11,25). Another longitudinal study among public health workers found Euroimmun ELISA responses increased in the first month and then decreased until week 12-16, with predicted proportion of sero-reverting of 59% after 12 months while Roche ECLIA responses increased, with no sero-reversions at week 24 and predicted sero-reverting of <2% at 12 months (26).*

**Reviewer #2:**

This article is important to PLOS Global Public Health to communicate new points of view and health information about differents locations of Africa, especially related to life conditions. However, the submission declare several limitations, but their methodological and ethical designed research must be considerable too.

***Authors’ response:*** *We thank the reviewer for the positive assessment of our study.*

It is an important research for Africa epidemiology studies but some some gaps were observed:
1. The study would might be correlated with studies of genomic sequence that might be help to correlate with specific variants.

***Authors’ response:*** *We thank the reviewer for this suggestion. For this study no genomic sequence data was available. In Lubumbashi, the study took place before the circulation of the Delta variant (which was dominant during the third wave). In Abidjan, a part of the survey took place after introduction of the delta variant. We clarified in the Discussion section:*

*Line 275: “We present here two of the first COVID-19 studies that combine mortality and seroprevalence surveys, providing mortality estimates and seroprevalence of anti-SARS-CoV-2 antibodies in a representative sample of the general population after the second pandemic wave in Lubumbashi, before the circulation of the Delta variant, and during and after the third pandemic wave in Abidjan during which the Delta variant circulated (13,14).”*

2. Lines 89 to 94 – “A detailed description of the study design and sampling methodology is provided in the supplementary information. The mortality questionnaire consisted of sections covering housing characteristics, demographic data, and information on deaths that occurred during the recall period. For households included in the seroprevalence survey, individual questionnaires were administered to each household member or their parent/guardian covering socio-demographic information, medical history, potential SARS-CoV-2 exposures, history of any possible COVID-related symptoms since 2020 and COVID-19 vaccination status.” Is it possible present any results of medical history, potential SARS-CoV-2 exposures, history of any possible COVID-related symptoms since 2020 from data collected?

***Authors’ response:*** *We thank the reviewer for this suggestion. We reported for both studies the proportion of individuals reporting COVID related symptoms:*

*Line 257: “The weighted proportion reporting COVID-19 related symptoms among RDT positive individuals was 22.4% (95%CI 16.2-29.5) in Lubumbashi and 71.6% (95%CI 65.5-77.2) in Abidjan. This proportion was similar among ELISA/ECLIA positive individuals (Lubumbashi 19.0% [95%CI 15.2-23.3]; Abidjan 71.8% [95%CI 65.7-77.4]).”*

*We now additionally state that we did not find significant positive associations of seropositivity with potential exposures such as social contact or work related contact and provide summary tables in the supplementary material:*

*Line 261: “We did not find significant positive associations of seropositivity and work related or social contact related exposures (Tables S17 and S18).”*

3. Is it possible explain increased malaria deaths in Pandemic period? (Table S1 and S2).

***Authors’ response:*** *We thank the reviewer for this suggestion. An increase in deaths due to other seasonal diseases such as malaria cannot completely be excluded. However we performed a sensitivity analysis to account for potential seasonal effects. For Lubumbashi, we compared the baseline period (01 January- 12 April 2020) to the exact same time period in 2021 during the pandemic (01 January- 12 April 2020). For Abidjan, we compared the period of the third wave (01 July 2021 - 10 November 2021), where an increase was detected, to its equivalent pre-pandemic period (01 July 2019 - 10 November 2019). All sensitivity analyses showed consistent results with the main findings.*

*We now mention the sensitivity analysis in the main manuscript:*

*Line 212: We performed a sensitivity analysis to investigate if observed increases during the pandemic period in Lubumbashi and the 3rd wave in Abidjan may have been caused by seasonal effects. The results of the sensitivity analysis showed consistent results with the main analysis (Supplementary tables 7 and 8).*

*And we specified in the limitations section:*

*Line 315: Moreover, we were not able to adjust for potential remaining confounding such as seasonality of other circulating pathogens. A sensitivity analyses restricting the analysis to the same months during the pre-pandemic and the pandemic phase showed however consistent results.*

*And provide the details of the sensitivity analysis in the Supplementary material:*

2. ***Sensitivity analysis – potential seasonal effects***

*To account for potential seasonal effects, for Lubumbashi, we compared the baseline period (01 January-12^th^ April 2020) to the equivalent time period in 2021 (01 January-12 April 2020) (Table S7).*

*For Abidjan, we compared the third wave to its equivalent pre-pandemic period (Table S8). Moreover, we restricted the baseline period to 01 January-12 April 2020 (as for Lubumbashi) to test for any introduced bias due to the longer baseline period in Abidjan (Table S8).*

*The results are consistent with the main analysis.*

***Table S7.*** *Sensitivity analysis for seasonal effects, Lubumbashi.*

| ***Time period*** | ***N deaths*** | ***CMR per 10 000 per day (95%CI)*** | ***Rate ratio (95%CI)*** | ***p-value*** |
| --- | --- | --- | --- | --- |
| ***Equivalent time periods pre-pandemic & pandemic periods*** | | | | |
| *Pre-pandemic*  *(01Jan20-12Apr20)* | *17* | *0.08 (0.05-0.14)* | *Baseline* | *NA* |
| *Pandemic equivalent*  *(01Jan21-12Apr21)* | *53* | *0.32 (0.24-0.42)* | *3.8 (2.1-6.9)* | *P<0.001* |

***Table S8.*** *Sensitivity analysis for seasonal effects, Abidjan.*

| ***Time period*** | ***N deaths*** | ***CMR per 10 000 per day (95%CI)*** | ***Rate ratio (95%CI)*** | ***p-value*** |
| --- | --- | --- | --- | --- |
| ***Equivalent time periods pre-pandemic & wave 3*** | | | | |
| *Pre-pandemic equivalent*  *(01July19-10Nov19)* | *7* | *0.04 (0.02-0.08)* | *Baseline* | *NA* |
| *Wave 3*  *(01July21-10Nov21)* | *11* | *0.11 (0.06-0.20)* | *3.0 (1.0-8.5)* | *p=0.043* |
| ***Shorter pre-pandemic (as in Lubumbashi)*** | | | | |
| *Pre-pandemic short*  *(01Jan20-12Apr20)* | *6* | *0.05 (0.02-0.13)* | *Baseline* | *NA* |
| *Pandemic*  *(13Apr20-10Nov21)* | *54* | *0.07 (0.05-0.09)* | *1.3 (0.5-3.2)* | *p=0.573* |

4. Lines 292-294 - "In addition, our results show that the infection has spread as much in Yopougon as in Marcory, in contrast to surveillance system figures which indicated a much lower attack rate in Yopougon". Is it possible clarify more detailed this affirmation?

***Authors’ response:*** *Thank you for the suggestion. We have clarified in the text to highlight that while we observed similar seroprevalence in Yopougon and Marcory, the ratio of infections to officially notified cases was much higher in Yopougon than Marcory.*

*Line 300: “Our seroprevalence results in Abidjan show similar spread in Yopougon as in Marcory, in contrast to surveillance system figures which indicated a much lower attack rate in Yopougon. In Abidjan, based on the ECLIA results from the 2nd phase and official notified cases, we estimated 1 in 31 infections were reported in Marcory and only 1 in 151 in Yopougon.”*

5.Is possible to add instruments of "mortality questionnaire (...) and of individual questionnaires(...)" lines 92-94?

***Authors’ response:*** *We now provide the generic questionnaire tools in the Supplementary material 2 that were slightly adapted to the specific study locations.*
